# Predator cues reduce intraspecific trait variability in a marine dinoflagellate

**DOI:** 10.1186/s12898-017-0119-y

**Published:** 2017-02-27

**Authors:** Sylke Wohlrab, Erik Selander, U. John

**Affiliations:** 1 Department of Ecological Chemistry, Alfred Wegener Institute, Helmholtz Centre for Polar and Marine Research, 27570 Bremerhaven, Germany; 2 Department of Biological and Environmental Sciences, University of Gothenburg, Vasaparken, 40530 Gothenburg, Sweden; 3 Helmholtz Institute for Functional Marine Biodiversity (HIFMB), 26111 Oldenburg, Germany

**Keywords:** *Alexandrium*, Saxitoxin gene expression, Grazer induced defense, Intraspecific trait variation, Predator–prey interaction

## Abstract

**Background:**

Phenotypic plasticity is commonplace and enables an organism to respond to variations in the environment. Plastic responses often modify a suite of traits and can be triggered by both abiotic and biotic changes. Here we analysed the plastic response towards a grazer of two genotypes of the marine dinoflagellate *Alexandrium fundyense,* evaluated the similarity of this response and discuss potential strain-specific trade-offs. We compared the expression of the known inducible defensive traits paralytic shellfish toxin content, and chain length. The effectiveness of the induced defense was assessed by monitoring grazing rates in both strains.

**Results:**

Our results show that the grazer cues diminish phenotypic variability in a population by driving the phenotype towards a common defended morphotype. We further showed that the expression of the *sxt*A gene that initiates the paralytic shellfish toxin biosynthesis pathway does not correlate with an observed increase in the paralytic shellfish toxin analogue saxitoxin, and that toxin induction differs in its physiological characteristics in both strains.

**Conclusion:**

Induced defense response in *Alexandrium* thus can directly affect further species interactions by reducing phenotypic variation and can result in genotype-dependent ecological trade-offs.

**Electronic supplementary material:**

The online version of this article (doi:10.1186/s12898-017-0119-y) contains supplementary material, which is available to authorized users.

## Background

Organisms can change their traits and thus their phenotype in response to variations in the environment. However, the ability of various genotypes to produce a common, environmentally cued phenotype may differ in terms of trade-offs associated with its expression caused by genetic variation between individuals [1]. In addition, the induction of one phenotype may indirectly influence the expression of linked traits and expose those traits to selection [2].

Phytoplankton cell size is a master trait that impacts growth, metabolism, and access to resources and therefore shapes the ecological niches of phytoplankton [3]. Cell size and related morphological traits like shape, and coloniality are extremely plastic traits that depend on many environmental variables such as light levels and nutrient concentrations [3]. Response to grazing pressure and related grazer resistance is significantly correlated with morphological traits as most grazers are confined to limited ranges of prey sizes [4]. The grazer’s ability to detect, capture, and handle the prey sets the lower limit of size whereas morphological e.g. constrains on handling larger prey set the upper limit [4]. For example, colony forming and size plasticity to escape grazing pressure is common in both marine (e.g. *Phaeocystis globosa*) and freshwater (e.g. *Scenedesmus*) phytoplankton [5]. The marine diatom *Skeletonema marinoi* adjust its chain length in the presence of copepod grazers towards smaller chain sizes but chain length was maintained in cultures exposed to microzooplankton grazers [6]. The bacterium *Flectobacillus* sp. induces filament formation when grazed by the microflagellates as single suspended cells were highly vulnerable to grazing, whereas filamentous cells were resistant to grazing [7].

Here we analysed the plastic response of two genotypes of the marine dinoflagellate *Alexandrium fundyense* in response to grazers or grazer cues. The genotypes were different in basal level of paralytic shellfish toxin production, amount of cells in chains, and ability to produce lytic extracellular substances, traits that are considered defensive. *Alexandrium* spp. are marine dinoflagellates and constitute together with diatoms and haptophytes the dominant groups of marine phytoplankton that accounts for approximately half of the global annual net primary production [8]. More than 80% of the marine phytoplankton production is consumed by herbivores, thus grazing is the most important loss factor for phytoplankton [9–11]. Grazing consequently exerts a strong selective pressure on phytoplankton and structures phytoplankton communities [12]. *Alexandrium* combines colony size plasticity and reduced swimming speed to avoid encounters with copepod grazers [13]. In addition, *Alexandrium* produces paralytic shellfish toxins (PSTs) in response to chemical cues from certain copepods [14–19]. Genotypes within a single natural *Alexandrium* population showed phenotypic differences concerning the production of PSTs and further uncharacterized allelochemical compounds [20, 21]. Such an intraspecific trait variation can alter predator–prey dynamics and can lead to altered gene frequencies within a population as a result of strong grazing pressure. Induced defense as response towards grazing yet can maintain genotypic diversity, however it may also bear different trade-offs associated with the inducible defense response for each genotype [1, 22].

Our aim was to investigate genotype dependent plastic responses in *Alexandrium* in order to characterize the effect of grazer cues on trait variability. The two genotypes investigated here differed under standard culture condition in the amount of PSTs produced, the number of cells in colonies, the ability to produce lytic compounds and showed genetic variation in terms of gene expression patterns [23]. We monitored the known inducible traits toxin content and chain length and investigated grazing rates of the copepods. The enhanced PSP-toxin production as response towards copepods and their cues seems to be an induction rather than an accumulations as e.g. caused by phosphorous limitation, We therefore analysed a potential direct correlation between the expression of the *sxtA* gene that initiates the saxitoxin biosynthesis pathway in *Alexandrium* and enhanced toxin production.

## Methods

### *Alexandrium fundyense* strains and zooplankton collection

The two clonal strains of *A. fundyense* Balech 1985 emended Anderson [24]; (before named as *Alexandrium tamarense*) were isolated from the North Sea coast east off Scotland [21] and grown in K-medium [25] in a temperature and light controlled room (salinity ~33, 18 °C, 14 h:10 h light–dark cycles, ~150 fmol m^−2^ s^−1^). The two strains, hereafter named Alex2 and Alex5 (both belong to the same population of the North American clade/ribotype group 1) are both producers of PSTs. Alex2 is further characterized by the presence of allelochemically active, unknown lytic compound(s) [20] whereas strain Alex5 lacks this ability.

Female *Centropages typicus* copepods were collected with vertical work package 2 (WP2) net hauls (200 µm mesh size) from ~20 m depth to the surface in the Gullmars fjord on the Swedish west coast. Only adult females were used in the experiments to minimize the variability between treatments. The copepods were maintained in the laboratory in filtered seawater (0.2 µm; salinity ~33) and fed *Rhodomonas baltica* (from the University of Gothenburg Marine Culture Collection, GUMACC) until the start of the experiments.

### Direct grazing experiments

For both *A. fundyense* strains 18 bottles with 500 mL (~5 × 10^6^ cells L^−1^) cultures in K/10 medium [25] was prepared. Half of the bottles received 10 *C. typicus* females per bottle, and the remaining bottles were kept as copepod free controls. All bottles were incubated in a temperature and light controlled room (salinity ~33, 18 °C, 14 h:10 h light–dark cycles, ~150 fmol m^−2^ s^−1^). Three replicates of the grazed and control treatments were harvested after 12, 48, and 72 h (see “sampling procedure” section below). Clearance rates were calculated using the equations of Frost [26].

### Cage experiments

The experiments with fed or starved copepods kept in cages were conducted in 500 mL glass bottles with cages made out of 50 mL polypropylene tubes with a 10 µm plankton mesh at the bottom and incubation conditions as described above. The plankton mesh constrained the organisms to their compartment (flask or cage) while allowing waterborne-cues to move between the compartments. For each *A. fundyense* strain, 9 bottles were set up consisting of three replicates of copepod-free controls, fed *C. typicus*, and starved *C. typicus*. Each bottle received 450 mL of *A. fundyense* culture with ~4 × 10^6^ cells L^−1^ in K/10 medium [25]. Cages were deployed into the flasks and filled with 30 mL of the same culture for the control treatments and the fed treatments. The flasks for the starved treatment received 30 mL of K/10 medium instead. Each cage of the fed and starved treatments received 10 *C. typicus* individuals i.e. the same amount as in the direct grazing experiment. The cages were gently moved up and down 5 times a day in order to promote the exchange of chemical cues between the compartments. The experiments were terminated after 48 h.

### Sampling procedure

Bottles were sealed and carefully turned over 7 times to ensure equal mixing of the culture. The direct grazing experiment samples were pre-filtered through a submerged 64 µm nylon mesh to remove copepods and copepods eggs. 60–70 mL were sub-sampled for enumeration and sizing of cells and chains using a coulter counter (Micromeritics, Norcross, USA) mounted with a 100 µm orifice tube and continuous stirring. A known volume of the subsamples was then suction filtered onto glass-fibre filters (Whatman) and stored at −20 °C until toxin extraction and analysis.

The remaining culture was concentrated on a 10 µm nylon mesh to collect the *A. fundyense* cells. The 10 µm mesh was rinsed into a 50 mL centrifuge tube with sterile filtered seawater and immediately centrifuged at 4 °C for 5 min for RNA samples. The supernatant was discharged and the pellet immediately mixed with 1 mL 60 °C hot TriReagent (Sigma-Aldrich, Steinheim, Germany) and transferred to a cryovial with acid washed glass beads. The cryovial was vortexed for 10 s and submerged into liquid nitrogen. The samples were stored at −80 °C until RNA isolation.

### PST analysis

Paralytic shellfish toxins samples (PST) were freeze-dried and extracted in 1 mL 0.05 M acetic acid (aq) through three consecutive freeze–thaw cycles. Extracts were filtered (GF/F) and stored frozen in glass vials. Samples were hydrolyzed with 0.1 M HCl at 100 °C for 10 min to transform C-toxins into their corresponding carbamates. We used a modified version of the method described by Asp and co-workers [27] to analyze all carbamate PSTs in a single run [16]. HPLC analysis was carried out on a Hitachi-7000 system equipped with a Genesis C8 column, (Vymac, 4 μm, 150*3 mm). A gradient between elution with 2 mM L^−1^ sodiumheptanesulfonate in 10 mM L^−1^ ammonium-phosphate buffer (pH 7.1) and 2 mM L^−1^ sodiumheptanesulfonate in 30 mM L^−1^ ammonium phosphate buffer (pH 7.1): acetonitrile (96:4) was used to separate PSTs. After the separation, toxins were oxidized with 7 mM periodic acid in 50 mM sodium phosphate buffer (pH 9.0) in a PEEK capillary (10 m, 80 °C). The oxidation was terminated with 0.5 M acetic acid before fluorescent detection at λ_ex_ = 330 nm, λ_em_ = 390 nm. Toxin standards were obtained from the certified reference materials program, National Research Council, Halifax, Canada.

### Total RNA isolation

TriReagent fixed and frozen cells were lysed using a Bio101 FastPrep instrument (Thermo Savant Illkirch, France) at maximum speed (6.5 m s^−1^) for 2 × 45 s. Lysed cells were cooled on ice, 200 µL chloroform was added and vortexed for 20 s. The samples were transferred to a phase lock tube after 5 min incubation at room temperature (Eppendorf, Hamburg; Germany) and incubated for another 5 min followed by centrifugation for 15 min at 13,000×*g* and 4 °C. The upper aqueous phase was transferred to a new tube and mixed with the same volume isopropanol, 1/10 volume of 3 M Na-acetate (pH 5.5; Ambion by Life Technologies, Carlsbad, California, USA) and 2 µL linear polyacrylamide (Ambion). Total RNA was precipitated for 90 min at −20 °C and collected by centrifugation for 20 min and 13,000×*g* at 4 °C. The obtained pellet was washed twice, first with 1 mL 70% EtOH followed by 1 mL EtOH absolute; the RNA pellet was dried for 1 min at 37 °C and resolved in 30 µL RNase free water (Qiagen, Hilden). RNA quality check was performed using a NanoDrop ND-1000 spectrometer (PeqLab, Erlangen, Germany) for purity and the RNA Nano Chip Assay with the 2100 Bioanalyzer device (Agilent Technologies, Santa Clara, California, USA) was just to examine the integrity of the extracted RNA. Potential DNA contaminations of RNA samples were tested by amplification of the 28 s rDNA gene. We considered our samples to be free of DNA as the respective gene could be amplified in positive controls only (containing both, RNA and added DNA).

### Expression analysis of the putative *sxt*A gene via RT-qPCR

The quantitative expression of the putative *sxt*A gene in Alex2 was evaluated via RT-qPCR using the method described in Freitag et al. [28]. For this RT-qPCR analysis, we only considered treatments were a significant increase in saxitoxin on the total PST profile compared to control cultures was observed (Alex2, direct grazing experiment, 48 and 72 h). Prior to cDNA synthesis, 500 ng of total RNA of the samples of interest were spiked with 10 ng of MA mRNA and 1 ng of NSP mRNA that served as an external Ref. [28]. The cDNA was synthesized with the Omniscript RT kit (Qiagen, Hilden, Germany) according to the manufacturer’s instructions with anchored oligo (dT) 20 primers. The synthesized cDNAs were diluted 1:5 and 2 µL were used to analyze the expression of the putative *sxt*A gene, the MA gene and the NSP gene with a SYBRgreen assay according to the manufactures protocol (Applied Biosystems/Life Technologies, Carlsbad, California, USA). Primers for the putative *sxt*A gene fragment had the sequences: 5′GCGAGACCGACGAGAAGTTC′3 and 5′AGCCGCTTGCGCTGAAG′3; primer sequences for the MA and NSP gene are given by Freitag et al. [28]. The qPCR reaction were carried out in a StepOnePlus™ Real-Time PCR System-device (Applied Biosystem by Life Technologies, Carlsbad, California, USA) with the following cycling parameters: 10 min initial denaturation at 95 °C, 40 cycles of 95 °C for 15 s and 60 °C for 1 min. A melting curve analysis was performed at the end of the reactions to verify the formation of a single PCR product in each reaction. In addition, standard curves with 6 points and a serial dilution factor of 1:10 starting with 10 ng PCR product from each gene were also analyzed with the SYBRgreen assay using the same primers and parameters. PCR products for the standard curves for the *sxt*A gene were obtained from the prepared cDNAs and the following primers: 5′CCGCCATATGTGCTTGTTTG′3 and 5′AGCTCCCTGTACACCTCTGC′3. Expression ratios were calculated using the equations of Pfaffl [29].

## Results

### Size distribution

**Fig. 1:**
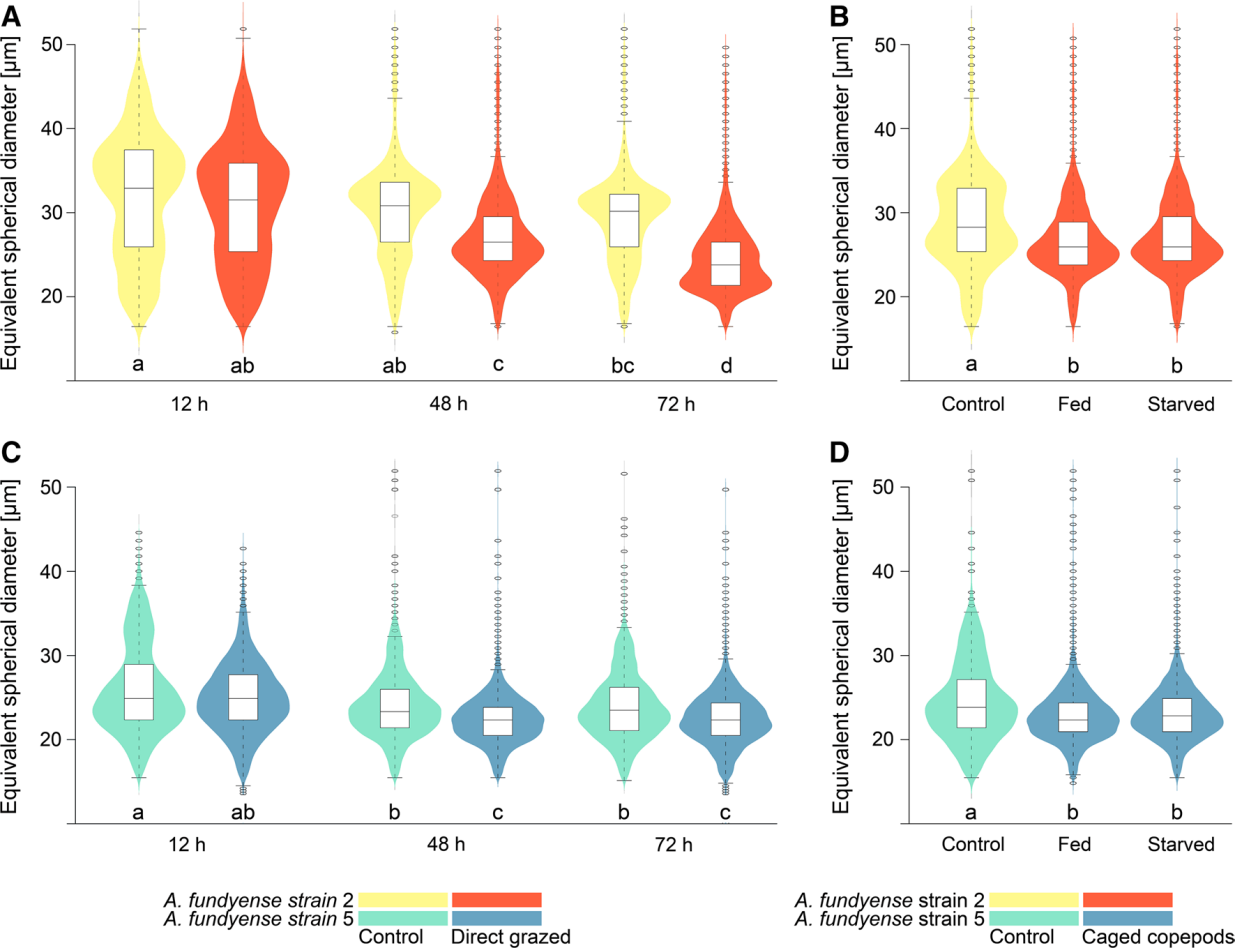
Cell size distribution of the *Alexandrium fundyense* strains. Cell size distributions of Alex2 (**A**, **B**) and Alex5 (**C**, **D**) after exposure to direct grazing *Centropages typicus* (**A**, **C**) or after exposure to waterborne-cues from caged fed and caged starved *C. typicus* (**B**, **D**). Treatments with waterborne-cues were terminated after 48 h. The diameter of the measured particles (equivalent spherical diameter in µm) is plotted as kernel density estimation with the width corresponding to the relative occurrence of a particle in a respective size class. *Box plots* show the 25th, 50th and 75th percentile; the ends of the whiskers mark the 95% intervals

The observed size distribution of the two strains of *A. fundyense* shifted in the presence of copepods towards a smaller equivalent spherical diameter (Fig. 1; Additional file 1). This shift was mainly caused by a different proportion of the culture present as single cells, two, or four cell chains as observed by microscopic examination. The shift is present in both the direct grazing experiments and the waterborne-cue experiments, with caged copepods, showing that the observed responses were triggered by the waterborne-cues from the copepods. The response was, however, strongest for Alex2 exposed to direct grazing copepods (Fig. 1A). Here, the average diameter significantly decreased by 10% after 48 h and by 16% after 72 h in grazer exposed cultured (two-way ANOVA *p* < 0.05, TukeyHSD *p* < 0.05). This decrease corresponds to approximately a doubling in the amount of singles cells compared to controls. The mean diameter of Alex2 cells exposed to waterborne-cues of caged fed and starved copepods significantly decreased by ~7% (one-way ANOVA *p* < 0.05, TukeyHSD *p* < 0.05) (Fig. 1B; Additional file 1).

Alex5 was generally less prone to form chains (Fig. 1C, D). Yet, exposure to both direct grazing and grazer cues resulted in a significantly reduced average size (mean diameter) also for this strain (~4% after 48 h and ~6% after 72 h, two-way ANOVA *p* < 0.05, TukeyHSD *p* < 0.05) (Additional file 1).

Different sampling times led to observable variations of cell size distributions in both strains (Fig. 1). Cells sampled at the time point 12 h have been collected just before the onset of the dark cycle that triggers cell division, and therefore also contain several cells that have accumulated enough biovolume to divide. Cells sampled at time points 48 and 72 h yet have been sampled after the dark cycle in the morning after cell division, thus showing a less scattered distribution with smaller overall cell sizes.

In the treatment with waterborne cues from caged fed and caged starved copepods, the cell size means significantly decreased by ~6.5% (one-way ANOVA *p* < 0.05, TukeyHSD *p* < 0.05) (Fig. 1D; Additonal file 1) showing that the response does not require any physical contact between copepods and algal cells.

### PST-contents and clearance rates

**Fig. 2:**
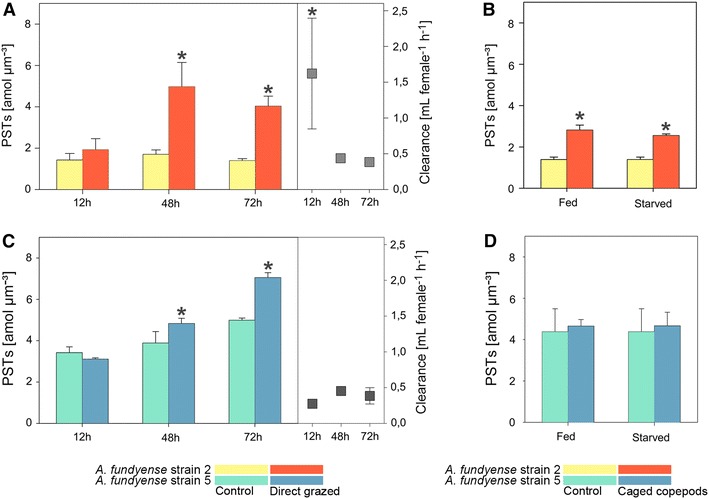
PST contents of the *Alexandrium fundyense* strains and clearance rates for *Centropages typicus*. PST contents of Alex2 (**A**, **B**) and Alex5 (**C**, **D**). PST contents after exposure to direct grazing *C. typicus* individuals and respective clearance rates are given in **A** and **C**. PST contents after exposure to waterborne-cues from caged fed and caged starved *C. typicus* are given in **B** and **D**. Treatments with waterborne-cues were terminated after 48 h. *Bars* marked with an *asterisk* show significant differences compared to either the control at this time point (for PSTs contents) or between the treatments at different time points (for clearance rates) (ANOVA *p* < 0.05)

Exposure of both *Alexandrium* strains to direct grazing *C. typicus* individuals resulted in significantly increased PST contents per biovolume in Alex2 and Alex5 (two-way ANOVA *p* < 0.05, TukeyHSD *p* < 0.05, Fig. 2A, B).

The treatment with waterborne-cues from caged fed and starved copepods however showed significant differences in the mean PST content for Alex2 only (one-way ANOVA *p* < 0.05, Fig. 2B), where PST content increased compared to the control (TukeyHSD *p* < 0.05).

Clearance rates on Alex2 significantly decreased from 1.7 mL per female per hour to 0.4 mL per female per hour (one-way ANOVA *p* < 0.05, Fig. 2A) during the direct grazing experiment. Clearance of copepods grazing on Alex5 cultures, however, remained constant around 0.4 mL per female per hour (Fig. 2C).

### PST profiles and differential expression of the *sxt*A gene

**Fig. 3:**
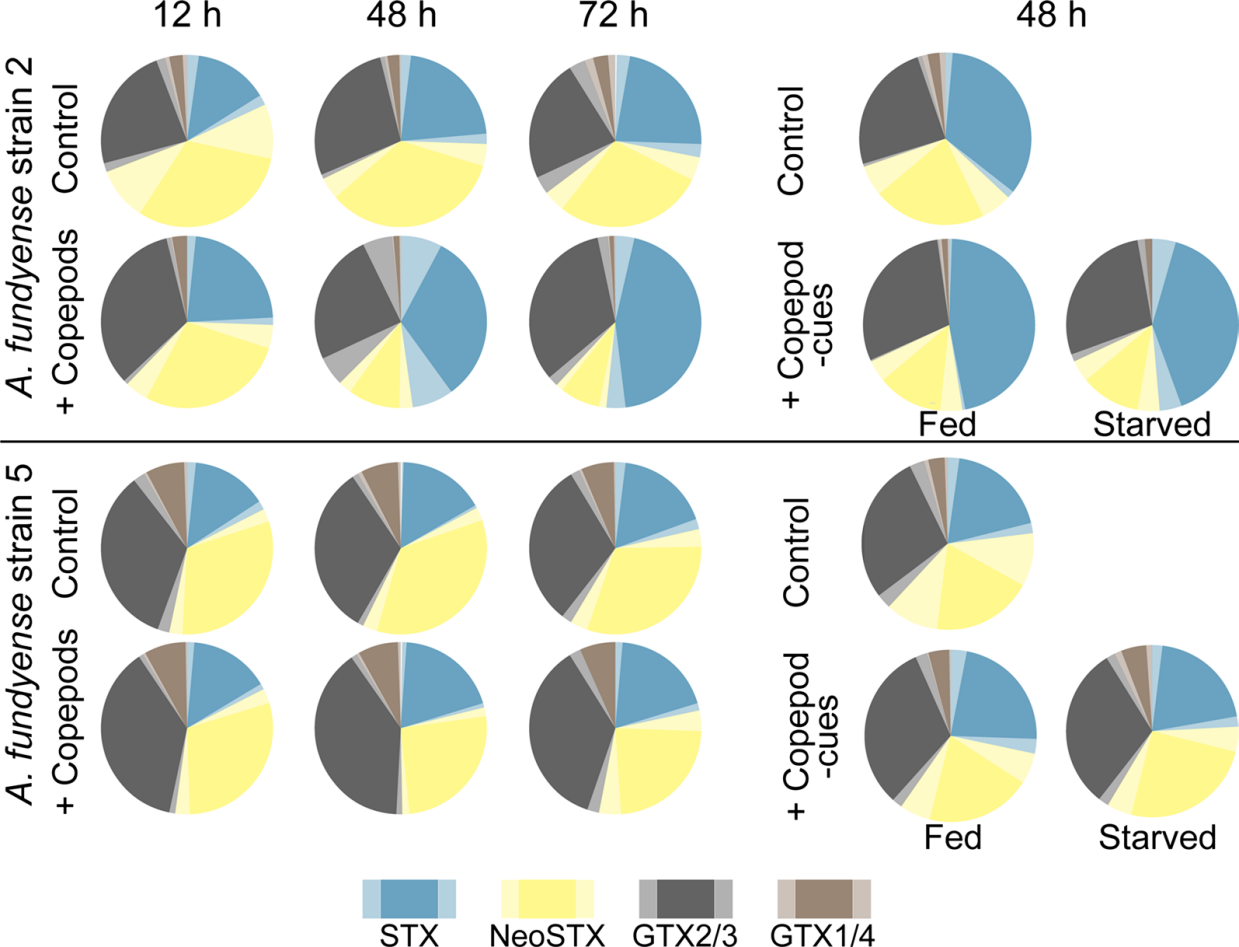
PST profiles of Alex2 and Alex5. PST profiles of Alex2 after exposure to direct grazing *Centropages typicus*, waterborne-cues of *C. typicus* and the respective controls are shown in the *upper panel*. PST profiles of Alex5 after exposure to direct grazing *C. typicus*, waterborne-cues of *C. typicus* and the respective controls are shown in the *lower panel*. *Shaded colors* indicate confidence intervals

**Fig. 4:**
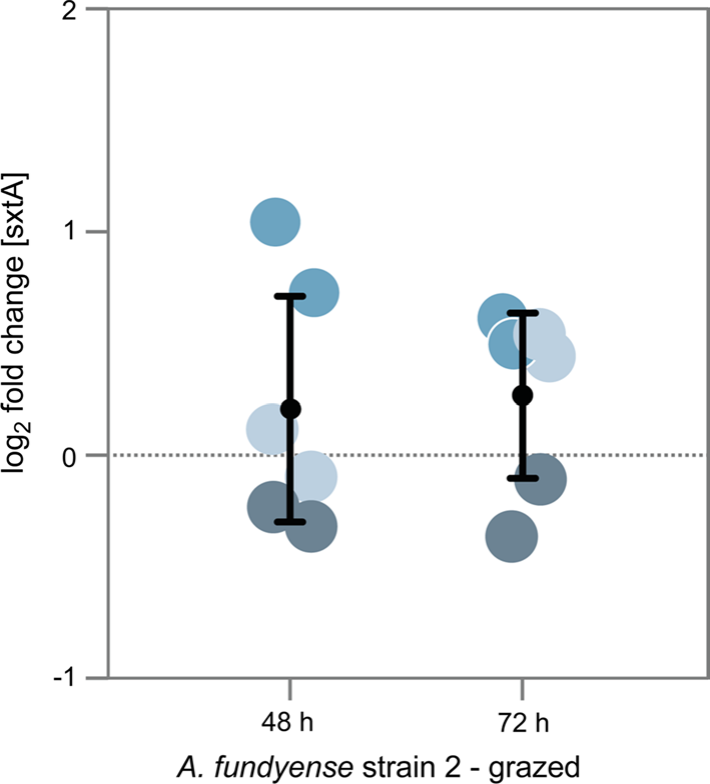
Expression of the *sxt*A gene fragment. The log2 expression ratio for the putative *sxt*A gene fragment as determined for Alex2 from the direct grazing experiment after 48 and 72 h. Quantification of the relative expression compared to the control treatments was done using a reference spike-in gene to normalize expression level of controls and treatments. The different *blue colours* represent biological replicates, *dots* with *same colours* represent technical replicates

The increase in the total PST content in Alex2 cultures after exposure to copepods in the direct grazing treatment and after exposure to caged fed and starved copepods was accompanied by a shift in the PST profile (Fig. 3). Yet, this phenomenon was not observed for Alex5. In Alex2, the amount of saxitoxin in the grazed treatment increased significantly from a mean of ~26% of total PSTs to 48% at 48 h and further to 52% at 72 h (ANOVA *p* < 0.05, TukeyHSD *p* < 0.05). The observed increase in the amount of saxitoxin in Alex2 did not result in a significant higher expression of the *sxt*A gene neither after 48 h nor at 72 h (Fig. 4).

## Discussion

The phenotypic differences show that Alex5 expresses the ‘defended’ phenotype, with smaller units (cells and/or cell chains) with higher toxin content already before exposure to grazers or grazer cues (Figs. 1, 2). Contrarily, Alex2 exhibited a less defended phenotype, with more cells in chains and lower toxin content in the absence of grazers or grazer cues. This was also accompanied by significant differences in the clearance rate of the copepods on the two genotypes. The clearance rates during the initial 12 h were fourfold lower for Alex5 compared to Alex2 (Fig. 2A, C). However, the grazing rates on Alex2 decrease to the same level after the induction of the defended phenotype (48 h, Figs. 1, 2). The phenotype expressed by Alex2 prior to defense induction is less vulnerable to unicellular heterotrophic grazers [20] and the allelochemicals produced by this strain did not seem to protect them against grazing copepods. Hence, the consequence of grazer exposure for Alex2 is a phenotypic shift towards less vulnerability towards copepod grazing. The splitting of cell chains in Alex2 may cause ecological costs under natural conditions i.e. a reduction of the ability to perform vertical migrations to retrieve nutrients at depth [13] and to resist unicellular grazers and competitors due to a reduced amount of cells in chains accompanied by a decreased concentration of allelochemicals surrounding the cells of this strain [30, 31]. These ecological costs may not only be strain-specific as they can indirectly influence the survival of conspecifics protected by the allelochemical cloud of this strain [23, 32]. Yet, the ability of *A. fundyense* to induce defense at different levels (morphological and physiological, as well as behavioural (see also Selander et al. [13]), offers flexibility in the applied defense strategy and allows to lower the predation risk at feasible costs depending on trade-offs that are themselves dependent on the environment and genotype [33, 34].

The increase in toxin biosynthesis differs in its physiological characteristics between the two strains: Alex5 increased the amount of PSTs without changing the relative composition of individual PSTs; in Alex2 the PST profile changed to a higher proportion of the saxitoxin derivate (Fig. 3). The higher amount of PST and in particular saxitoxin in Alex2 did, however, at the measured time points not correlate with a higher expression of the putative *sxt*A gene [35] that initiate the saxitoxin biosynthesis pathway as verified via RT-qPCR (Fig. 4). The *sxt*A gene product might not be the limiting step in the biosynthesis of saxitoxin or might have been upregulated already at the onset of the induced defense response prior to the observed morphological and physiological changes. Further, the biosynthesis of saxitoxin could be regulated by other means e.g. post-transcriptional, by the regulation of other enzymes participating in this pathway [36] or by the provision of precursors and reduction equivalents. The observed differences in the two strains may also point towards a convoluted biosynthesis pathway where several routes could lead to an increase in intracellular PST content. An increase in PSTs could therefore be as well associated with changes in other traits where beneficial trait associations led to a selective advantage. For example, we cannot rule out that the PST increase per biovolume is purely due to a decrease of cell size in our experiment. However, previous studies with the same genotype Alex5 showed increased PST contents after 48 h exposure to copepods on a per cell basis, irrespective of the biolvolume [17]. Selander et al. showed that after removal of copepods, grazer-induced PST contents in *A. fundyense* returned to control levels after approximately 5 cell divisions, whereas the average biovolume returned to control levels after one to two cell divisions. Thus *A. fundyense* cells remained more toxic compared to controls with comparable biovolumes [16].

With the cage experiment design we demonstrated that the copepod cues alone are sufficient to induce the expression of defensive features. However, due to a potential signal dampening effect the signals alone lead to a weaker response compared to the direct grazing experiments (Figs. 1, 2) [17, 30, 37, 38]. The reduction of cell chains, however, remained at comparable levels to the direct grazing experiment in both strains (Figs. 1, 2). A meta-analysis on herbivore resistance in plants presumed that traits other than secondary metabolites (e.g. morphology, life history, phenology, primary chemistry and physiology) are more effective in defense [39]. As the effect of increased toxin content in *Alexandrium* on grazing rates strongly depends on the grazer abundance, species composition and local adaptations [37, 40–45], the significance of increased toxin content for defense is still unclear, particularly because intoxication of the grazer benefits the whole population and additionally potential competitors, unless the more toxic cells are rejected by grazers and directly benefit from their inducible toxicity [15, 37, 41]. The observed morphological changes are preventive by reducing encounter rates with grazers [13] and may therefore directly contribute increased fitness to responding cells.

## Conclusions

In conclusion, we showed that the presence of copepods has the potential to induce the formation of a comparable phenotype from initially different genotypes which highlights the selective pressure that emanates from grazers in the pelagic realm. We could further show that the morphological changes might be stronger correlated to the induced defense response than changes in PST-content are. The induced defense response will reduce the trait variability for natural selection to operate on; the costs for its induction however can be different for each strain on several levels i.e. in terms of physiological and ecological costs. Selection on different genotypes can therefore occur in the presence of plasticity due to different costs and trade-offs that arise for the respective genotype. The occurrence of multiple environmental cues to respond to in nature however might equalize the effects of strain-specific trade-offs within populations.

## Additional file

**Additional file 1.** Information about the measured cell size distributions.

## Abbreviation

PST(s): paralytic shellfish toxin(s)

## References

[1] Bolnick DI, Amarasekare P, Araújo MS, Bürger R, Levine JM, Novak M, Rudolf VHW, Schreiber SJ, Urban MC, Vasseur DA (2011). Why intraspecific trait variation matters in community ecology. Trends Ecol Evol.

[2] Pfennig DW, Wund MA, Snell-Rood EC, Cruickshank T, Schlichting CD, Moczek AP (2010). Phenotypic plasticity’s impacts on diversification and speciation. Trends Ecol Evol.

[3] Litchman E, Klausmeier CA (2008). Trait-based community ecology of phytoplankton. Annu Rev Ecol Evol Syst.

[4] Hansen B, Bjornsen PK, Hansen PJ (1994). The size ratio between planktonic predators and their prey. Limnol Oceanogr.

[5] Van Donk E, Ianora A, Vos M (2011). Induced defences in marine and freshwater phytoplankton: a review. Hydrobiologia.

[6] Bergkvist J, Thor P, Jakobsen HH, Wängberg S-Å, Selander E (2012). Grazer-induced chain length plasticity reduces grazing risk in a marine diatom. Limnol Oceanogr.

[7] Corno G, Jürgens K (2006). Direct and indirect effects of protist predation on population size structure of a bacterial strain with high phenotypic plasticity. Appl Environ Microbiol.

[8] Falkowski PG, Katz ME, Knoll AH, Quigg A, Raven JA, Schofield O, Taylor FJR (2004). The evolution of modern eukaryotic phytoplankton. Science.

[9] Calbet A (2001). Mesozooplankton grazing effect on primary production: a global comparative analysis in marine ecosystems. Limnol Oceanogr.

[10] Calbet A, Landry MR (2004). Phytoplankton growth, microzooplankton grazing, and carbon cycling in marine systems. Limnol Oceanogr.

[11] Cyr H, Pace ML (1993). Allometric theory-extrapolations from individuals to communities. Ecology.

[12] Verschoor AM, Vos M, van der Stap I (2004). Inducible defences prevent strong population fluctuations in bi- and tritrophic food chains. Ecol Lett.

[13] Selander E, Jakobsen HH, Lombard F, Kiørboe T (2011). Grazer cues induce stealth behavior in marine dinoflagellates. Proc Natl Acad Sci..

[14] Selander E, Kubanek J, Hamberg M, Andersson MX, Cervin G, Pavia H (2015). Predator lipids induce paralytic shellfish toxins in bloom-forming algae. Proc Natl Acad Sci.

[15] Selander E, Thor P, Toth G, Pavia H (2006). Copepods induce paralytic shellfish toxin production in marine dinoflagellates. Proc R Soc B-Biol Sci.

[16] Selander E, Fagerberg T, Wohlrab S, Pavia H (2012). Fight and flight in Dinoflagellates? Kinetics of simultaneous grazer-induced responses in *Alexandrium tamarense*. Limonol Oceanogr.

[17] Wohlrab S, Iversen MH, John U (2010). A molecular and co-evolutionary context for grazer induced toxin production in *Alexandrium tamarense*. PLoS ONE.

[18] Yang I, Selander E, Pavia H, John U (2011). Grazer-induced toxin formation in dinoflagellates: a transcriptomic model study. Eur J Phycol.

[19] Senft-Batoh CD, Dam HG, Shumway SE, Wikfors GH (2015). A multi-phylum study of grazer-induced paralytic shellfish toxin production in the dinoflagellate *Alexandrium fundyense*: a new perspective on control of algal toxicity. Harmful Algae.

[20] Tillmann U, Hansen P (2009). Allelopathic effects of *Alexandrium tamarense* on other algae: evidence from mixed growth experiments. Aquat Microb Ecol.

[21] Alpermann TJ, Tillmann U, Beszteri B, Cembella AD, John U (2010). Phenotypic variation and genotypic diversity in a planktonic population of the toxigenic marine dinoflagellate *Alexandrium tamarense* (Dinophyceae). J Phycol.

[22] Tillmann U, Alpermann TL, da Purificação RC, Krock B, Cembella A (2009). Intra-population clonal variability in allelochemical potency of the toxigenic dinoflagellate *Alexandrium tamarense*. Harmful Algae.

[23] Wohlrab S, Tillmann U, Cembella A, John U. Trait changes induced by species interactions in two phenotypically distinct strains of a marine dinoflagellate. ISME. 2016. 10.1038/ismej.2016.57 PMC5113847 27093044

[24] John U, Litaker RW, Montresor M, Murray S, Brosnahan ML, Anderson DM (2014). Formal revision of the *Alexandrium tamarense* Species Complex (Dinophyceae) taxonomy: the introduction of five species with emphasis on molecular-based (rDNA) classification. Protist.

[25] Keller MD, Selvin RC, Claus W, Guillard RRL (1987). Media for the culture of oceanic ultraphytoplankton. J Phycol.

[26] Frost BW (1972). Effects of size and concentration of food particles on the feeding behavior of the marine planktonic copepod *Calanus pacificus*. Limnol Oceanogr.

[27] Asp TN, Larsen S, Aune T (2004). Analysis of PSP toxins in Norwegian mussels by a post-column derivatization HPLC method. Toxicon.

[28] Freitag M, Beszteri S, Vogel H, John U (2011). Effects of physiological shock treatments on toxicity and polyketide synthase gene expression in *Prymnesium parvum* (Prymnesiophyceae). Eur J Phycol.

[29] Pfaffl MW (2001). A new mathematical model for relative quantification in real-time RT-PCR. Nucleic Acids Res.

[30] Harvell DC, Tollrian R, Tollrian R, Harvell DC (1999). Why inducible defenses?. The ecology and evolution of inducible defenses.

[31] Jonsson PR, Pavia H, Toth G (2009). Formation of harmful algal blooms cannot be explained by allelopathic interactions. Proc Natl Acad Sci USA.

[32] John U, Tillmann U, Hülskötter J, Alpermann TJ, Wohlrab S, Van de Waal DB (2015). Intraspecific facilitation by allelochemical mediated grazing protection within a toxigenic dinoflagellate population. Proc R Soc Lond B: Biol Sci..

[33] Ellner SP (2013). Rapid evolution: from genes to communities, and back again?. Funct Ecol.

[34] Bjærke O, Jonsson PR, Alam A, Selander E (2015). Is chain length in phytoplankton regulated to evade predation?. J Plankton Res..

[35] Stüken A, Orr RJS, Kellmann R, Murray SA, Neilan BA, Jakobsen KS (2011). Discovery of nuclear-encoded genes for the neurotoxin saxitoxin in dinoflagellates. PLoS ONE..

[36] Perini F, Galluzzi L, Dell’Aversano C, Iacovo E, Tartaglione L, Ricci F, Forino M, Ciminiello P, Penna A (2014). SxtA and sxtG gene expression and toxin production in the Mediterranean *Alexandrium minutum* (Dinophyceae). Marine Drugs.

[37] Senft-Batoh CD, Dam HG, Shumway SE, Wikfors GH, Schlichting CD (2015). Influence of predator–prey evolutionary history, chemical alarm-cues, and feeding selection on induction of toxin production in a marine dinoflagellate. Limnol Oceanogr.

[38] Bergkvist J, Selander E, Pavia H (2008). Induction of toxin production in dinoflagellates: the grazer makes a difference. Oecologia.

[39] Carmona D, Lajeunesse MJ, Johnson MTJ (2011). Plant traits that predict resistance to herbivores. Funct Ecol.

[40] Turner JT, Tester PA (1997). Toxic marine phytoplankton, zooplankton grazers, and pelagic food webs. Limnol Oceanogr.

[41] Teegarden GJ (1999). Copepod grazing selection and particle discrimination on the basis of PSP toxin content. Mar Ecol Prog Ser.

[42] Turner TJ, Doucette JG, Powell LC, Kulis MD, Keafer AB, Anderson MD (2000). Accumulation of red tide toxins in larger size fractions of zooplankton assemblages from Massachusetts Bay. USA. Mar Ecol Prog Ser.

[43] Colin SP, Dam H (2004). Testing for resistance of pelagic marine copepods to a toxic dinoflagellate. Evol Ecol.

[44] Teegarden GJ, Campbell RG, Anson DT, Ouellett A, Westman BA, Durbin EG (2008). Copepod feeding response to varying *Alexandrium* spp. cellular toxicity and cell concentration among natural plankton samples. Harmful Algae.

[45] Dam HG, Haley ST (2011). Comparative dynamics of paralytic shellfish toxins (PST) in a tolerant and susceptible population of the copepod *Acartia hudsonica*. Harmful Algae.

