# Supplementary material for: Predator cues reduce intraspecific trait variability in a marine dinoflagellate
Source: BMC Ecol. 2017 Feb 27;17:8. doi: 10.1186/s12898-017-0119-y (PMC5327569; doi:10.1186/s12898-017-0119-y)
Supplement: Supplementary file 1 — Additional file 1. Information about the measured cell size distributions. [file 12898_2017_119_MOESM1_ESM.pdf]

Additional file 1: Measured size classes of the *Alexandrium fundyense* strains Alex2 and Alex5

***A. fundyense* strain**	**Treatment / time**			**25th percentile**	**50th percentile**	**75th percentile**
Alex2	Direct grazing	48 h	Control	26.5 µm	30.8 µm	33.6 µm
			Grazed	24.3 µm	26.5 µm	29.5 µm
		72 h	Control	25.9 µm	30.2 µm	32.2 µm
			Grazed	21.3 µm	23.8 µm	26.5 µm
	Waterborne-cues	48h	Control	25.4 µm	28.3 µm	32.9 µm
			Fed	23.8 µm	25.9 µm	28.9 µm
			Starved	24.3 µm	25.9 µm	29.5 µm
Alex5	Direct grazing	48 h	Control	21.3 µm	23.3 µm	25.9 µm
			Grazed	20.4 µm	22.3 µm	23.8 µm
		72 h	Control	21.3 µm	23.8 µm	26.5 µm
			Grazed	20.4 µm	22.3 µm	24.3 µm
	Waterborne-cues	48h	Control	21.3 µm	23.8 µm	27.1 µm
			Fed	20.9 µm	22.3 µm	24.3 µm
			Starved	20.9 µm	22.8 µm	24.8 µm
